# Precrop Functional Group Identity Affects Yield of Winter Barley but Less so High Carbon Amendments in a Mesocosm Experiment

**DOI:** 10.3389/fpls.2018.00912

**Published:** 2018-07-03

**Authors:** Richard van Duijnen, Julien Roy, Werner Härdtle, Vicky M. Temperton

**Affiliations:** ^1^Institute of Ecology, Leuphana University of Lüneburg, Lüneburg, Germany; ^2^Institut für Biologie, Ökologie der Pflanzen, Freie Universität Berlin, Berlin, Germany; ^3^Berlin-Brandenburg Institute of Advanced Biodiversity Research, Berlin, Germany

**Keywords:** crop rotation, arbuscular mycorrhizal fungi, rhizobia, barley, high carbon amendment, immobilization, plant functional group, nitrate leaching

## Abstract

Nitrate leaching is a pressing environmental problem in intensive agriculture. Especially after the crop harvest, leaching risk is greatest due to decomposing plant residues, and low plant nutrient uptake and evapotranspiration. The specific crop also matters: grain legumes and canola commonly result in more leftover N than the following winter crop can take up before spring. Addition of a high carbon amendment (HCA) could potentially immobilize N after harvest. We set up a 2-year mesocosm experiment to test the effects of N fertilization (40 or 160 kg N/ha), HCA addition (no HCA, wheat straw, or sawdust), and precrop plant functional group identity on winter barley yield and soil C/N ratio. Four spring precrops were sown before winter barley (white lupine, faba bean, spring canola, spring barley), which were selected based on a functional group approach (colonization by arbuscular mycorrhizal fungi [AMF] and/or N_2_-fixing bacteria). We also measured a subset of faba bean and spring barley for leaching over winter after harvest. As expected, N fertilization had the largest effect on winter barley yield, but precrop functional identity also significantly affected the outcome. The non-AMF precrops white lupine and canola had on average a positive effect on yield compared to the AMF precrops spring barley and faba bean under high N (23% increase). Under low N, we found only a small precrop effect. Sawdust significantly reduced the yield compared to the control or wheat straw under either N level. HCAs reduced nitrate leaching over winter, but only when faba bean was sown as a precrop. In our setup, short-term immobilization of N by HCA addition after harvest seems difficult to achieve. However, other effects such as an increase in SOM or nutrient retention could play a positive role in the long term. Contrary to the commonly found positive effect of AMF colonization, winter barley showed a greater yield when it followed a non-AMF precrop under high fertilization. This could be due to shifts of the agricultural AMF community toward parasitism.

## Introduction

An ever-increasing yield is necessary to feed the growing world population, but this is coupled with high fertilizer use and associated environmental problems (Matson, 1997). Nitrate leaching is one of these problems, especially in intensive agriculture (Nixon and European Environment Agency, 2003), leading to multiple negative effects such as eutrophication of surface waters, or pollution of drinking water with consequences to human health (Di and Cameron, 2002). In temperate agroecosystems, the most crucial time point for leaching to occur is in the fall and winter, when crop residues decompose, and plant nitrogen uptake and evapotranspiration is low (Di and Cameron, 2002). Certain management practices to avoid nitrate leaching at this time point have been tested, such as addition of a substrate with a high C/N ratio (high carbon amendment; HCA) in an attempt to immobilize the nitrate microbially. The rationale behind this is that microbes in soils are commonly C-limited (Kallenbach and Grandy, 2011; Farrell et al., 2014), and by adding easily available carbon the microbes will take up the excess carbon and simultaneously immobilize excess mineral nitrogen. The advantage of this concept has been tested various times already with mixed success in mechanistic incubation studies (Zavalloni et al., 2011; Congreves et al., 2013a) and field studies (Thomsen and Christensen, 1998; Vidal and López, 2005; Burke et al., 2013; Congreves et al., 2013b; Török et al., 2014), for both agricultural and restoration purposes. However, due to the complexity of soil nitrogen dynamics, it is not clear whether remineralization of the immobilized N takes place the following spring, thus bridging the high leaching risk period in fall/winter and providing nitrogen when plant uptake is high (Chaves et al., 2007).

The amount of nitrogen susceptible to leaching in fall also depends on the previous crop (from now on referred to as ‘precrop’). This can largely be affected by the crop type. Cereals like wheat have relatively low leftover N and risk of N leaching (Maidl et al., 1991; Francis et al., 1994), whereas N-intensive crops with a deep rooting system, such as canola (*Brassica napus*), or vegetable crop residues typically have very high leftover N (Henke et al., 2008; Agneessens et al., 2014). Similarly, grain legumes can increase leaching risk due to easily decomposable high-N plant residues (Chalk, 1998; Plaza-Bonilla et al., 2015). However, in the case of legumes as a precrop, of which benefits commonly have been attributed to a more positive N balance due to atmospheric N fixation (Maidl et al., 1996; Herridge et al., 2008), the overall effects of legumes in crop rotations cannot solely be attributed to increased N benefits. Reduced soil-borne pathogens, reduced soil water usage, and deep tap root systems loosening the soil can also positively affect the next crop (Peoples et al., 2009). This generally results in a yield increase compared to non-legumes in cereal cropping systems (Chalk, 1998; Angus et al., 2015).

Besides the positive effect of legumes in crop rotation, precrops that form a symbiosis with arbuscular mycorrhizal fungi (AMF), thus providing a host, typically increase the AMF spores in the soil and colonization of the next (mycorrhizal) crop. Although the benefits of AMF are usually linked to increased phosphorus and water uptake, there might also be benefits to N uptake, although this matter is still open to question (Smith and Smith, 2011; Thirkell et al., 2016), and disease resistance (Cameron et al., 2013). However, most studies neglect the possible role of AMF in affecting yields, in contrast to the well-studied effects of management practices, such as fertilizer addition and tillage, on subsequent crop yields. One reason for this might be that both fertilizer levels (especially P), tilling depth and the extent of fallow periods generally negatively affect AMF performance (Lekberg and Koide, 2005; Fester and Sawers, 2011). Moreover, these intensive agricultural practices could indirectly select for AMF strains which do not provide the benefits to the host species, but instead are closer to the parasitic end of the spectrum, investing more in their own reproduction and maximizing carbon acquisition from the host plants (Ryan and Graham, 2002; Verbruggen and Kiers, 2010). However, it is not clear what effect non-mycorrhizal crop species (the most common ones belong to the *Brassicaceae)* in crop rotations have on AMF community and structure both during the cropping with the non-mycorrhizal plant species and for the subsequent mycorrhizal crop (Kirkegaard et al., 2008; Verbruggen and Kiers, 2010). We know little about the extent to which having a mycorrhizal vs. a non-mycorrhizal precrop affects the yield and performance of the subsequent crop.

The effect and applicability of HCAs to counter nitrate leaching might depend on the specific precrop. To this end, we combine precrops with an ecologically based plant functional group approach based on two common plant-microbe symbioses: colonization by AMF and/or N_2_-fixing bacteria. We explore the role of the symbiotic status of the precrop by combining all possible combinations of these two symbioses in the precrop, e.g., from rhizobial and mycorrhizal to non-rhizobial and non-mycorrhizal species (see **Table 1**). We include high and low N fertilization to disentangle effects of precrop functional groups and HCAs and their interactions. To our knowledge, this is the first study that explicitly tests the role of such plant functional groups (based on symbiosis) with a full factorial design within an agricultural experiment. Therefore, our study incorporates an ecological concept within a mainly agricultural experimental setup.

**Table 1:** Sowing and harvest dates, fertilizer amount and symbioses with AMF and/or rhizobia of the crops used in this study.

	Crop	Sowing date	Harvest date	Fertilizer addition (kg/ha)					AMF	Rhizobial
				N	K_2_O	P_2_O_5_	MgO	S		
Precrops	Spring barley	26/05/16	26/08/16	75	130	40	35	98	X	
	Spring canola	26/05/16	22/09/16	100	140	70	50	90		
	Faba bean	26/05/16	07/09/16	0	50	115	35	60	X	X
	White lupine	26/05/16	28/09/16	0	50	60	35	65		X
Focal crop	Winter barley	07/10/16	10/07/17	160/40	100	70	50	86	X	

*Precrop complete fertilizer was added at precrop sowing date, while complete fertilizer addition to winter barley occurred at 23/03/17, except for two more N additions at 01/05/17 and 15/05/17 in the high N treatment.*

We experimentally investigated the role of HCA, precrop functional group and nitrogen fertilization on winter barley yield in an outside mesocosm experiment. We measured a subset, consisting of faba bean and spring barley as precrops, for the effect of these precrops and HCA on nitrate leaching over winter. We asked the following questions:

(1) Does the previous crop identity affect winter barley yield and do precrops forming root symbioses (rhizobia/AMF) show a bigger positive effect under low N?
(2) Is the effect of HCA on winter barley yield modulated by N fertilization level?
(2) Is the effect of HCA affected by the precrop identity? More specifically, does nitrate leaching increase after harvest of a legume precrop compared to a non-legume precrop and is this reduced by HCAs?

Overall, we hypothesized that an AMF-colonizing legume precrop amended with wheat straw under high N conditions results in the highest winter barley yield. Specifically, we hypothesized that:

(1a) Legume precrops have a positive effect on winter barley yield (especially under low N fertilization), since they introduce extra N into the system.
(1b) AMF crops have a positive effect compared to non-AMF crops on winter barley yield; we expect the highest yield increase with faba bean (both rhizobial and AMF).
(2) HCA has no effect under optimal N conditions, but could either decrease or increase winter barley yield under low N conditions by continuous N-immobilization or N-immobilization followed by remineralization in the spring, respectively.
(3a) Precrop identity modifies HCA effects on winter barley yield, since we expect more leftover mineral N after harvest of the legumes and canola. This would lead to potentially higher N immobilization over winter.
(3b) Nitrate leaching after precrop harvest is higher for a legume precrop compared to a non-legume precrop (faba bean vs. spring barley) and decreases for both with HCA addition.

## Materials and Methods

### Experimental Site and Conditions

Our mesocosm experiment was conducted outside in an experimental garden of the University of Lüneburg (Lüneburg, Germany, 53°14′23.8″N 10°24′45.5″E). Mean annual temperature and rainfall is 9.2°C and 718 mm respectively. For detailed meteorological measurements during the experiment see Supplementary Figure S1, data was taken from the nearby weather station of the Deutsche Wetterdienst, Wendisch Evern (53°12′49.0″N 10°28′13.1″E).

### Experimental Design

**FIGURE 1:**
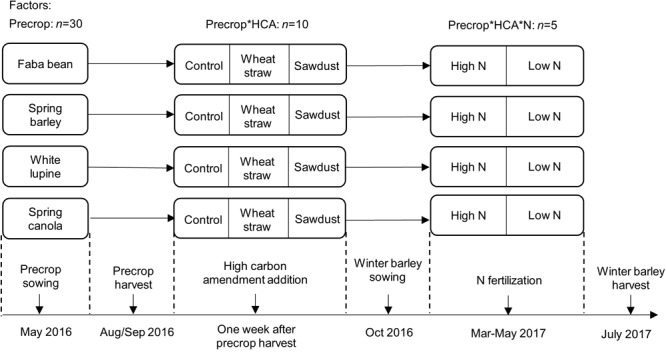
Experimental design of the study with the main factors, precrops, HCA and winter barley N fertilization, added at different time points. For exact sowing and harvest time points see **Table 1**.

We applied a mesocosm experiment to quantify treatment effects on winter barley yield. We used relatively square mesocosms with an edge length of 37.5 and 26.5 cm at the top and the bottom, respectively, and a height of 37 cm; the resulting volume was 38 L. We used a surface area of 0.16 m^2^ when converting to g m^-2^ and calculating fertilizer and HCA rates from kg ha^-1^. Mesocosms were subject to three experimental factors (**Figure 1**): HCA (three levels; no HCA, wheat straw, sawdust), precrop identity (four levels; spring barley, spring canola, faba bean, white lupine), and N fertilization (2 levels; high: 160 kg/ha, low: 40 kg/ha). We applied a full factorial design with 5 replicates (*n* = 5) for each treatment combination, resulting in 120 mesocosms. Mesocosms were placed randomly (with 25 cm distance between mesocosms) in the experimental garden. Mesocosms were filled to a bulk soil density of ∼1.1 g cm^-3^ in the top 10 cm with soil passed through a 1 cm sieve. The soil originated from the top 0–30 cm of the experimental farm Hohenschulen of the Christian-Albrechts-University in Kiel (54°19′05.6″N 9°58′38.8″E). The soil is a sandy loam (Cambic Luvisol) and has a history of agricultural practice. In the growing season before the experimental start, a mixture of catch crops (such as clover and lupine) had been grown without fertilization, while the season before that maize had been cultivated and fertilized with 40 m^3^ slurry (∼3% N, ∼1.8% P) and 100 kg/ha triple superphosphate (20% P). The soil had a total of 1.26% C, 0.14% N, a C/N ratio of 9.2 and a pH of 6.0 at the start of the experiment. We constructed a setup to measure nitrate concentrations in the leachate after the precrop harvest until N fertilization of winter barley at 23/03/2017 (see subsection leachate measurements). After the precrop harvest, mesocosms were reorganized and six out of ten replicates of the precrops faba bean and barley, and all HCAs were randomly selected for leachate measurements. At this time point, mesocosms were also isolated with air cushion foil (Luftpolsterfolie 3S, Hermann Meyer KG) and covered with white plastic sheets to avoid extreme temperature fluctuations within the mesocosm.

### Study Species and Crop Husbandry

In May 2016, all mesocosms were sown with the precrops, which were chosen according to their ability to either be colonized by AMF or rhizobia (i.e., *Fabaceae*). The chosen precrops were spring barley (*Hordeum vulgare* cv. Barke, Saatzucht Breun), spring canola (*Brassica napus* cv. Medicus, NPZ), faba bean (*Vicia faba* cv. Tiffany, NPZ) and white lupine (*Lupinus albus* cv. Energy, Feldsaaten Freudenberger). Winter barley (*Hordeum vulgare*, cv. Antonella, Nordsaat Saatzucht), the focal crop, was sown the season after the precrops. The planting density (seeds/m^2^) and row distance (cm, if applicable) was the following: spring barley 300, 9 cm; canola 120, 19 cm; faba bean 45; white lupine; 70, winter barley; 240, 13 cm. Mesocosms were fertilized according to their crop and standard agricultural practice in Germany (for exact values see **Table 1**) at either the sowing date (all precrops) or on 23/03/17 (winter barley). N Fertilization of winter barley for the high N treatment was spread over three time points, 60 kg N/ha at 23/03/2017 and 01/05/2017, and 40 kg N/ha at 15/05/2017. Nitrogen was added in the form of calcium ammonium nitrate, phosphate as superphosphate, potassium oxide as Korn-Kali, magnesium oxide as Korn-Kali and Epsom salt, and sulfur was contained in superphosphate, Epsom salt and Korn-Kali.

All mesocosms received 0.8 g Schneckenkorn (9.9 g/kg iron(III)-phosphate; Neudorff GmbH) on 14/06/2016 to counter plant damage by slugs. Furthermore, all mesocosms were sprayed with roughly 200 ml diluted Spruzit Schädlingsfrei per pot (45.9 mg/L pyrethrin; Neudorff GmbH) on 01/07/2016 due to an aphid infestation. No pesticides or herbicides were necessary during winter barley cultivation. Weeding was done by hand when necessary on multiple occasions. Mesocosms were watered during dry and warm spring/summer days when deemed necessary, but never during fall/winter, as to not affect the leachate amount.

### High Carbon Amendments

High carbon amendments were added within 1 week after the precrop harvest. HCAs were air-dry wheat straw or spruce sawdust at a rate of 8.6 t/ha (137.6 g/mesocosm). The C/N ratios were 71 and 539, total C 46 and 51%, and total N 0.7 and 0.1%, respectively. The wheat straw had a particle size of 5–10 cm, whereas the sawdust contained particles of 1–2 cm. The HCAs were mixed in the top 10 cm of the soil and afterwards watered slightly to promote incorporation. The top 10 cm soil in the control treatment was also mixed, but without any amendment added.

### Leachate Measurements

A leachate setup was built after the precrop harvest to collect water flowing through the mesocosms, which was subsequently analyzed for nitrate concentration. Mesocosm pots were put into slightly smaller mesocosms (30 × 30 × 32.5 cm) on top of two stacks of pallets. The drainage holes of the smaller mesocosms were sealed with cement and coated with a nitrogen-free resin at a slight angle so all water would flow to an attached drain. The drain was connected to a 5 L canister stored under the pallets and covered with white plastic sheet. Drainage holes of the large mesocosm were covered with nitrogen-free drainage fleece (Drainage-Geotextilvlies, Haga-Welt), to prevent contamination by soil particles or root growth into the smaller mesocosms. From 01/09/16 until 23/03/17, every 3–4 weeks (when enough water for analysis was leached through) the total leachate volume was recorded and a subsample of 50 ml was taken. The subsample was stored at -20°C before analysis for nitrate content. Samples were filtered before analysis using 0.45 μm filters (CHROMAFIL Xtra RC 45/25 membrane, Macherey-Nagel, Germany). The first two time points were analyzed with a direct UV measurement (VWR UV-3100PC, Denmark) at 220 nm and subtraction of interference at 275 nm according to (Goldman and Jacobs, 1961). However, at the third time point (28/11/16) the interference at 275 nm was too high (>10%) and we measured this and subsequent time points with an ion chromatograph (Dionex DX-120, AS14 column, United States). We correlated the UV and ion chromatograph for the third time point and the measured values showed good agreement (*R*^2^= 0.961; Supplementary Figure S2), but the UV method underestimated nitrate content due to the high interference at 275 nm. Three samples were excluded from the analysis due to broken tubes.

### Plant and Soil Analyses

All precrops were harvested at maturity in September/August 2016 (for exact dates see **Table 1**), winter barley at 10/07/17, and separated into seeds and other aboveground biomass tissues, i.e., stems and leaves. Stems were cut off at 3 cm above the ground and leftover stubble and roots remained in the mesocosm. Furthermore, the seeds, depending on the crop, were manually separated from the spike (spring and winter barley) or the pods (lupine, canola and faba bean) to get the final cleaned seed mass. Dry mass of each plant component was measured after drying for at least 48 h at 70°C to constant weight. Winter barley seeds free from spikes were milled (MM 400, Retsch, Germany), dried at 105°C overnight and analyzed for C and N content (Vario EL, Elementar, Germany). Soils were sampled for roots at precrop harvest for screening for AMF colonization to see if the potential for symbiosis actually resulted in a symbiotic interaction in the experiment. Pooled composite samples of 6 soil cores (0–10 cm depth, 1 cm diameter) per mesocosm were sieved at 0.5 mm and precrop root fragments were sampled and stored at -80°C until analyses. A subset of precrop root fragments were then stained with Trypan blue and screened for AMF structures.

Soils were sampled for C/N analysis on two separate occasions. From 13 till 15 March 2017, before nitrogen fertilization, a composite sample of 6 cores (0–10 cm depth, 1 cm diameter) per mesocosm was taken. Afterward, autoclaved soil of the start of the experiment was used to fill the holes, as to not interfere with the leachate setup. Sampled areas were marked with a wooden toothpick to avoid resampling the same position later on. After the winter barley harvest on 10 July 2017, a composite sample of again 6 cores (0–10 cm depth, 2 cm diameter) was taken. Samples were air-dried before sieving (2 mm), milling (MM 400, Retsch, Germany), drying 24 h at 105°C and subsequent C/N analysis (Vario EL, Elementar, Germany).

### Statistical Analysis

We first fitted three-way ANOVA models testing the effect of HCA, precrop and N fertilization as fixed factors on grain yield, straw biomass, C/N ratio and total N uptake of the seeds. The factor levels were as following; Precrop: spring barley, faba bean, white lupine, canola; HCA: control, wheat straw, sawdust, N: high, low. We included all interactions, because we were mainly interested in the two-way interactions between N fertilizer and either precrop species or HCA, i.e., whether the response to these factors differs between low and high N conditions. Moreover, we tested for the interaction between precrop and HCA, in case the HCA response was dependent on the precrop. We started with the full model and also checked for significance of the three-way interaction. If this was not significant and if dropping this improved the model, which was so in all cases, the three-way interaction was dropped. In case of no interaction, we averaged over the other factors for the factor of interest when plotting means or describing effect sizes.

In most cases heteroscedasticity was observed due to the choice of extremely contrasting N levels (i.e., high and low). Thus, for all data involving N as a factor, we used a generalized least square model with the “weights” function to allow different error terms for high and low N and correct for this heteroscedasticity [varIdent (form = ∼1|N)] using the nlme package (Pinheiro et al., 2017). The same approach was applied to soil C/N analysis, but replacing N levels with HCA levels within the weights function. Multiple comparisons between groups were tested for significance by using generalized linear hypotheses with Tukey’s HSD adjusted *p*-values using the lsmeans (Lenth, 2016) and multcomp (Hothorn et al., 2008) packages. The leachate data was analyzed with a two-way ANOVA testing the effect of HCA (control, wheat straw, sawdust) and precrop (faba bean, spring barley) on total nitrate leached from the precrop harvest in August/September 2016 until N fertilization in March 2017. Multiple comparisons were tested for significance using Tukey’s HSD adjusted *p*-values using the multcomp package (Hothorn et al., 2008). All statistical analyses were performed using R 3.4.2 (R Core Team, 2017).

## Results

### N Fertilization

N fertilization was the main factor affecting the yield of winter barley with an average increase of 75% at high N compared with the low N level. This main factor effect was expected and we were mainly interested in the interactions. We found strong interaction effects with N levels and precrop species for the yield parameters grain yield, straw biomass, seed C/N ratio and total seed N uptake (**Table 2**).

**Table 2:** Results of the GLS ANOVA of N fertilizer, precrop and HCA on different yield parameters.

Factor	df	Grain yield		Straw biomass		C/N seeds		Total N yield	
		*F*	*p*	*F*	*p*	*F*	*p*	*F*	*p*
N	1, 102	490.58	**<0.0001**	935.99	**<0.0001**	1017.73	**<0.0001**	1749.64	**<0.0001**
Precrop	3, 102	8.12	**0.0001**	9.37	**<0.0001**	0.31	0.8179	16.96	**<0.0001**
HCA	2, 102	11.05	**<0.0001**	10.69	**0.0001**	2.18	0.1186	8.55	**0.0004**
N^∗^precrop	3, 102	7.58	**0.0001**	6.60	**0.0004**	12.36	**<0.0001**	7.07	**0.0002**
N^∗^HCA	2, 102	1.43	0.2433	2.13	0.1239	0.37	0.6919	0.84	0.4355
Precrop^∗^HCA	6, 102	2.11	0.0589	2.70	**0.0179**	1.56	0.1655	1.33	0.2503

*ANOVA *p*-values are in bold when *p* < 0.05.*

### Precrop Species Affect Yield Under High N

**FIGURE 2:**
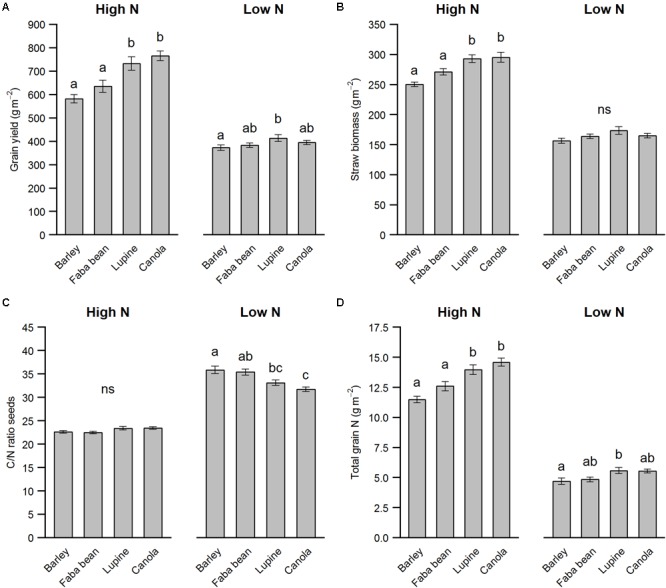
Grain yield parameters of winter barley as affected by nitrogen fertilization and precrop species. The parameters are grain yield **(A)**, straw biomass **(B)**, C/N ratio of the seeds **(C)** total grain N **(D)**. The values are means per factor combination averaged over HCA (*n* = 15) ± SE. Different letters indicate significant differences between groups within N levels (*p* < 0.05).

The precrop had a pronounced effect on the winter barley yield, but mostly under high N conditions only (**Figure 2A**, precrop^∗^N: *F*_3,102_ = 7.56, *p* < 0.001). Non-AMF precrops lupine and canola resulted in an on average 23% increase in yield compared to the AMF precrops spring barley and faba bean under high N. Although under low N we found significantly higher yields in winter barley when grown after lupine compared to spring barley, this effect was not as pronounced compared to the stimulating effect of having a non-mycorrhizal precrop found in the high N treatment (**Figure 2A**). For the winter barley straw yield, although roughly half the biomass of the yield in all treatments, the same pattern was found for high N but no significant difference found under low N (**Figure 2B**). Interestingly, the C/N ratio of the seeds showed the reverse pattern: Non-AMF precrops resulted in a lower seed C/N ratio than AMF precrops, but only under low N conditions (precrop^∗^N: *F*_3,102_ = 12.36, *p* < 0.001, **Figure 2C**). Total grain N uptake, however, which is the N concentration of the seeds multiplied by grain yield, showed the same pattern as the grain yield, indicating that the C/N ratio of the seeds is a less important indicator for total N uptake (**Figure 2D**). Although we had two legumes in the crop rotation, we did not see a clear legume effect on the yield, but instead observed a consistent effect of non-AMF vs. AMF precrops.

### Sawdust Decreases Yield, but Increases Soil C/N Ratio

**FIGURE 3:**
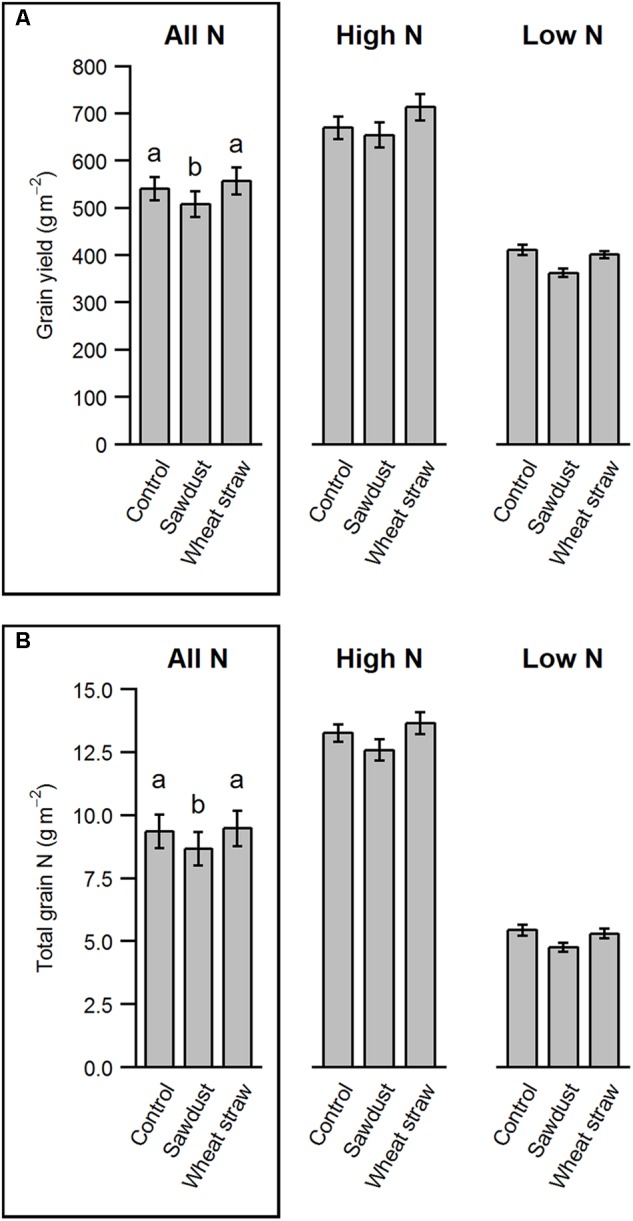
Grain yield parameters of barley as affected by HCA. The parameters are grain yield **(A)** and total grain N **(B)**. The values are means ± SE per factor combination averaged over N and precrop (*n* = 40) in the boxed panel and separately plotted for high and low N to show variability between N levels (*n* = 20). Different letters indicate significant differences between groups (*p* < 0.05).

Overall, HCA application did not result in a winter barley yield increase compared to the control treatment, irrespective of N fertilization (HCA: *F*_2,102_ = 11.05, *p* < 0.001; N^∗^HCA: *F*_2,102_ = 1.43, *p* = 0.243). However, sawdust application consistently decreased grain yield (sawdust: -6.3%, wheat straw: +3.8% compared to control, **Figure 3A**). Although seed C/N ratios were not affected by HCA (**Table 2**), total N uptake was lower in the sawdust treatment due to decreased yield (**Figure 3B**). Furthermore, we found a marginally significant HCA and precrop interaction on grain yield (precrop^∗^HCA: *F*_6,102_ = 2.11, *P* = 0.059). This was mostly seen in spring barley and faba bean causing a slightly increased yield and white lupine and canola slightly decreased yield when wheat straw was applied.

Top soil (0–10 cm) C/N ratios increased with HCA application, more so with sawdust than wheat straw (**Table 3**). Total C and corresponding C/N ratios increased with higher addition of carbon, but no change in total N was observed before N fertilization in March 2017. However, while wheat straw resulted in an increase of soil C/N ratios in March, no lasting effect compared to the control was found by the time of the winter barley harvest in July 2017. There is a trend that C/N and total C due to sawdust addition remained high even over the main growing season of winter barley (**Table 3**), although in our experimental setup this was statistically not testable due to addition of N over the sampling period. Lastly, we did not find an effect of any precrops on the measured soil parameters.

**Table 3:** Effect of HCA and N fertilization on soil C/N ratio, total C (%) and total N (%).

Sampling date	HCA	N	*n*	C/N	Total C (%)	Total N (%)
13/03/17	C		20	9.68 (0.0640)a	1.29 (0.009)a	0.134 (0.0005)ns
	W		20	10.08 (0.0628)b	1.36 (0.009)b	0.135 (0.0008)ns
	S		20	11.87 (0.2445)c	1.59 (0.033)c	0.134 (0.0007)ns
10/07/17	C	High	20	9.52 (0.071)a	1.29 (0.017)a	0.135 (0.0018)a
		Low	20	9.51 (0.046)a	1.26 (0.014)a	0.132 (0.0014)a
	W	High	20	9.70 (0.057)a	1.36 (0.015)b	0.141 (0.0018)b
		Low	20	9.60 (0.062)a	1.33 (0.014)b	0.139 (0.0011)b
	S	High	20	11.48 (0.164)b	1.60 (0.022)c	0.141 (0.0018)b
		Low	20	11.27 (0.203)b	1.53 (0.035)c	0.138 (0.0015)b
N effect				ns	*	*

*C: Control, W: Wheat straw, S: Sawdust. Sampling of March 2017 did not have the N treatment yet, thus a random subsample of low and high N factors was taken. Values are means ± SE averaged over precrops. Different letters indicate significant main effects of HCA (Tukey HSD; *p* < 0.05). Asterisks indicate a significant N effect (only applicable for sampling date 10/07/17).*

### Leachate

**FIGURE 4:**
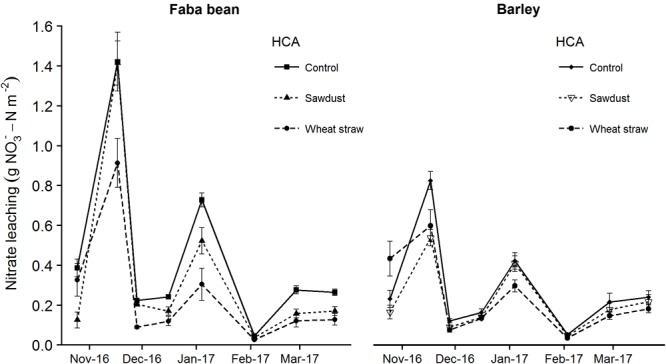
Nitrate leaching after precrop harvest until first N fertilizer application as affected for the precrops faba bean and spring barley, and HCA. Nitrate leaching is calculated as the concentration of a subsample times the volume of water leached through the mesocosm. The values are means ± SE (*n* = 4–6).

**FIGURE 5:**
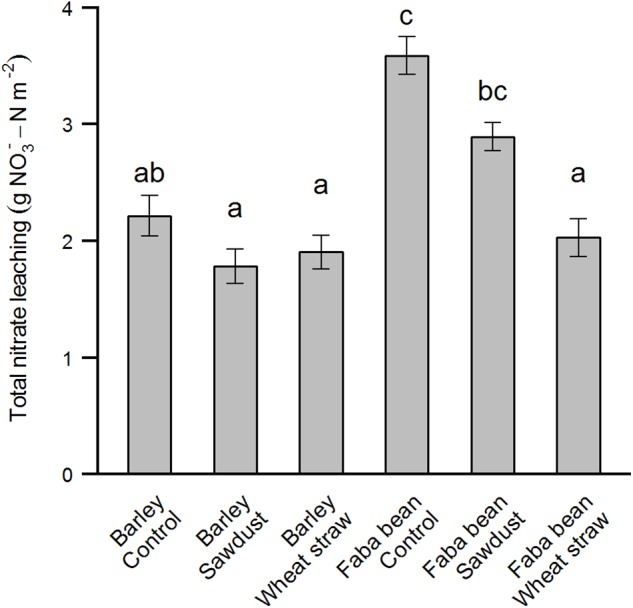
Total amount of nitrate leached between 01/09/16 and 21/03/17 as affected by the precrops faba bean and barley and HCA. Total nitrate leaching is calculated as the concentration of a subsample times the volume of water leached through the mesocosm summed over all time points between 25/10/16 and 21/03/17. The values are means ± SE (*n* = 4–6). Different letters indicate significant differences between groups (*p* < 0.05).

Measurement of nitrate leaching showed a clear trend of increased leaching when faba bean was grown as a precrop compared to barley (**Figures 4, 5**). The first sampling time point was 25/10/16, although we had the leachate sample setup ready 01/09/16, due to a very dry and warm September month (Supplementary Figure S1). Wheat straw showed a pattern of decreasing nitrate leaching for both precrops (**Figure 4**). For the cumulative nitrate leaching over fall and winter, faba bean, being a legume, had significant higher nitrate leaching in the control group (precrop^∗^HCA: *F*_2,27_ = 10.22, *P* < 0.001; **Figure 5**), but wheat straw addition reduced nitrate leaching by 43% compared to the control. Although wheat straw addition lowered faba bean leaching to values similar to barley as a precrop, there was no significant overall decrease in barley due to HCAs.

### AMF Colonization of Precrops

We did a screening of the roots of all four precrops to see if they showed signs of AMF or other fungal structures. Some fungal structures in these roots are shown in Supplementary Figure S4. We found that faba bean had vesicles, hyphae and spores resembling AMF structures, whereas we did not find clear signs of AMF colonization in spring barley. As expected, the non-AMF precrops canola and lupine showed no signs of AMF colonization.

## Discussion

We determined the response of winter barley yield to the previous crop and HCA under low or higher N conditions. We used a plant functional group approach based on two important plant–microbe symbioses (AMF and rhizobia) to disentangle their temporal effect on crop yield. Contrary to our hypotheses, we did not see a large effect of either plant functional group under N-limiting conditions. However, under high N fertilizer conditions non-AMF precrops significantly increased the yield compared to AMF precrops. Whereas HCA did not have a strong effect on the yield, it resulted in an increase in total soil C and N, indicating possible longer term positive effects on nutrient retention. HCA also directly reduced nitrate leaching in the top soil, but only for faba bean compared to spring barley as a precrop.

### Effect of Precrop and Its Type of Symbiosis on Winter Barley Yield

We hypothesized that precrops that are leguminous and/or have a symbiosis with AMF would positively affect winter barley yield, especially under low N conditions. Instead, we hardly found an effect under low N conditions and a positive effect of non-AMF precrops on barley yield under high N (**Figure 2A**). The legumes were clearly fixing atmospheric nitrogen, since (a) we found large numbers of nodules on the main tap roots when mixing in the HCA after precrop harvest, and (b) we found no signs of N stress, and legume yield comparable to canola and spring barley without any N fertilization (**Table 1** and Supplementary Figure S3). Nevertheless, our results suggest that the legume precrop effect was not the dominant driver for winter barley yield, but more that the AMF-symbiosis of the precrop played a key role, since winter barley yield after canola (non-AMF and non-rhizobial) was just as high as that after lupine (non-AMF, rhizobial). This result is surprising because of the many studies showing a positive effect of legumes on the subsequent crop in crop rotations (Chalk, 1998; Angus et al., 2015).

This lack of strong legume facilitation on the subsequent crop might be explained by our crop husbandry and experimental setup. First of all, we grew grain legumes until maturity and removed all of the aboveground biomass, (both stems and seeds) which may complicate a direct comparison to typical leguminous cover crops where the goal is to increase nutrient retention and add biologically fixed nitrogen in the system (Tonitto et al., 2006). However, just the legume grain alone can contribute to 45–75% of the total aboveground biomass N (Van Kessel and Hartley, 2000), thus normally the majority of N is taken off in grain legumes. In our study, any carry over N facilitation effect would have to be mediated via decomposition of roots or direct exudation of compounds. However, belowground N contributions to the N budget are often ignored or vary widely in their estimates (Herridge et al., 2008), especially in the case of rhizodeposition (Wichern et al., 2008).

Secondly, our mesocosms were only 37 cm deep, which limits the extrapolation to field conditions, since the roots of our species could not grow as deep as in field conditions. Canola and barley, and grain legumes similar to the species in our study such as narrow leaf lupine (*Lupinus angustifolius)* and soybean (*Glycine max)* are known to have roots as deep as 1.6, 1.7, 2.5, and 1.8 m, respectively (Canadell et al., 1996; Fan et al., 2016). Thus, one would expect such roots under field conditions to be able to take up more of the excess N before being lost out of the system as leachate. After harvest in winter, nitrate will leach down to lower soil depths (Pedersen et al., 2009), some of which may then be taken up by the next crop in spring, some of which will be lost as leachate. In our study, however, we measured leachate derived from a 37 cm deep mesocosm, such that one could not know whether the N would be lost in the same way as leachate under field conditions. Field experiments and models show large amounts of nitrate leaching into deeper soil layers (Pedersen et al., 2009). Leachate measurement in our study nevertheless allowed us to compare differences between a legume and non-legume precrop. We know that cropping systems with legume species tend to have a larger leachate problem than non-leguminous crops since legumes tend to not rely on soil N as much as other crops, and leave low residues with low C/N ratios (Francis et al., 1994; Hauggaard-Nielsen et al., 2009). Our direct finding that faba bean had higher nitrate leaching compared to spring barley as a precrop (**Figure 5**) confirms this.

In addition to a positive legume precrop effect, we expected a positive effect of AMF precrops on the winter barley yield. This was not the case in the high N treatment (**Figure 2A**) and a surprising finding, because, assuming a positive effect on mycorrhizal colonization when the previous crop is a host to AMF compared to a non-host, a higher AMF colonization is associated with a higher yield (Lekberg and Koide, 2005). However, due to inclusion of low and high N, we can rule out a significant N carry-over of the previous crop, because we found no legume-exclusive effect compared to non-legumes (spring barley or canola; **Figure 2A**). Thus, the non-AMF precrop effect under high N might be attributed to other factors, such as reduced AMF colonization or a reduction in soil-borne pathogens by bio-fumigation of canola (Matthiessen and Kirkegaard, 2006). However, a bio-fumigation effect would not explain the similar positive effect of lupine. Therefore, a decrease in winter barley yield due to AMF precrops might be the most plausible explanation. Root staining showed AMF colonization in faba bean roots, but no clear colonization in spring barley (in comparison, in canola or lupine we found other fungal structures, but no colonization by AMF; Supplementary Figure S4). We can therefore not say with certainty whether the negative precrop effect of spring barley on winter barley yield compared to canola or lupine is directly related to AMF performance.

Explicit comparisons, other factors being equal, between non-mycorrhizal and mycorrhizal crops in crop rotations are limited (Ryan and Graham, 2002; Lekberg and Koide, 2005). Although rather controversial, Ryan and Graham (2002) and Ryan and Kirkegaard (2012) question the function of AMF and their contribution to crop yields in intensive agriculture. Similarly in our experiment, nutrient conditions were standard for German agriculture, which is generally regarded as very high (de Vries et al., 2011; MacDonald et al., 2011). High fertilizer rate/soil nutrients, especially soluble P, is known to negatively affect AMF colonization (Mäder et al., 2000; Treseder, 2004), but could also change the functioning of the AMF community toward more parasitism (Verbruggen and Kiers, 2010). If the AMF community represented a typical agricultural community (due to the history of our soil), this could explain the positive effect of non-AMF precrops, with the AMF precrops possibly introducing rather parasitic AMF to the system that may have contributed to the lower yield in winter barley after these crops in our study.

### HCA Effects on Winter Barley and Soil Parameters

Addition of HCAs to reduce nitrogen leaching specifically after harvest has been attempted multiple times, with mixed results (Thomsen and Christensen, 1998; Vidal and López, 2005; Chaves et al., 2007; Congreves et al., 2013b). HCAs can have effects on a number of parameters. Some studies found effects on the soil chemistry (which is often the main focus), whereas effects on the subsequent crop performance are much rarer (Congreves et al., 2013b). This is surprising, since the preferred outcome of HCA N immobilization over winter would be to retain more N in the topsoil, thus making it more available to the next crop and reducing the N fertilizer needs of the subsequent crop.

In our study, we did not find strong evidence of remineralization of immobilized N due to HCA (**Table 3**). On the contrary, sawdust application had a negative effect on winter barley yield, potentially caused by strong N immobilization under either N fertilizer levels. Wheat straw application resulted in a positive trend of winter barley yield under higher N conditions (**Figure 3A**), which could be either caused by remineralization of immobilized N during the growing season, but also due to decomposition and subsequent N release contained in the wheat straw itself (Di and Cameron, 2002). We found no difference in total N content in soils in March 2017, which indicates a lack of N transfer over fall/winter, although at winter barley harvest we did find a significant increase of total N in the wheat straw and sawdust treatment compared to the control (**Table 3**). This increase might be mainly due to fertilizer added during the winter barley growing season being immobilized in the soil rather than an N carry-over effect from the precrop. It is worthwhile noting that because of the small particle size of sawdust some particles were not sieved out with a 2 mm sieve before milling, while pieces of wheat straw were, which could inflate the soil C measurement. However, the results were consistent and wheat straw also showed a higher total C (%) than the control (**Table 3**).

We found a strong reduction in nitrate leaching when wheat straw was applied to faba bean as a precrop (**Figure 5**). Other studies on HCAs and nitrate leaching show mixed results. Chaves et al. (2007) found a reduction in N leaching of 56–68% due to wheat straw or sawdust after high N vegetable crop residues, which might be comparable to the increased leftover N in legumes. On the other hand, little to no reduction in nitrate leaching due to straw incorporation was found by Thomsen and Christensen (1998) when cereal crops or sugar beet were grown beforehand, similar to our findings for spring barley as a precrop. A common finding in both these studies is that remineralization in the next spring does not seem to occur in considerable amounts. Paradoxically, HCAs could increase N leaching when immobilized N is being mineralized next fall instead. Finally, we did not find a precrop species effect on soil C/N ratios or total soil C or N contents in either March before N fertilization or at winter barley harvest, despite the clear reduction in nitrate leaching after faba bean amended with wheat straw. This could be because the mineral N pool is relatively small compared to the total N pool, and, coupled with the hypothesis of long-term immobilization due to HCAs, might explain the lack of a positive HCA effect, especially under low N conditions.

## Conclusion

Using a semi-natural setup our experiment bridged the gap between short-term artificial greenhouse experiments and the heterogeneity of field studies, allowing for relatively realistic weather conditions and temporal scale whilst reducing spatial heterogeneity, in order to improve our understanding of carry-over effects of precrops. We found evidence that AMF precrops had possibly parasitic effects on the subsequent winter barley when large amounts of fertilizer were added to the system, whereas there was no clear legume precrop effect. In our setup, short-term immobilization of N by HCA addition after harvest was not generally achieved, despite a slight positive effect of wheat straw on winter barley yield. HCAs do show potential to counter nitrate leaching of high-risk leaching crops such as grain legumes. Furthermore, other effects such as an increase in SOM or nutrient retention could play a positive role in the long term, since we found higher soil total C and total N nearly a year after application of HCAs.

## Author Contributions

VT and RvD designed the experiments. RvD and JR collected the data. RvD analyzed the data. RvD and VT led the writing. WH reviewed the manuscript. All authors contributed to critical revisions of the manuscript.

## Conflict of Interest Statement

The authors declare that the research was conducted in the absence of any commercial or financial relationships that could be construed as a potential conflict of interest.
