# Supplementary material for: Precrop Functional Group Identity Affects Yield of Winter Barley but Less so High Carbon Amendments in a Mesocosm Experiment
Source: Front Plant Sci. 2018 Jul 3;9:912. doi: 10.3389/fpls.2018.00912 (PMC6037990; doi:10.3389/fpls.2018.00912)
Supplement: Supplementary file 1 [file Data_Sheet_1.docx]

Supplementary Material

Precrop functional group identity affects yield of winter barley but less so high carbon amendments in a mesocosm experiment

**Richard van Duijnen^1^, Julien Roy^2,3^, Werner Härdtle^1^, Vicky M. Temperton^1*^**

^1^Institute of Ecology, Leuphana University Lüneburg, Lüneburg, Germany

^2^Institut für Biologie, Ökologie der Pflanzen, Freie Universität Berlin, Berlin, Germany

^3^Berlin-Brandenburg Institute of Advanced Biodiversity Research, Berlin, Germany

**Corresponding author:**Vicky M. Temperton
[vicky.temperton@leuphana.de](mailto:vicky.temperton@leuphana.de)

# Running title: Precrop and Carbon Supplementary Figures and Tables

## Supplementary Figures


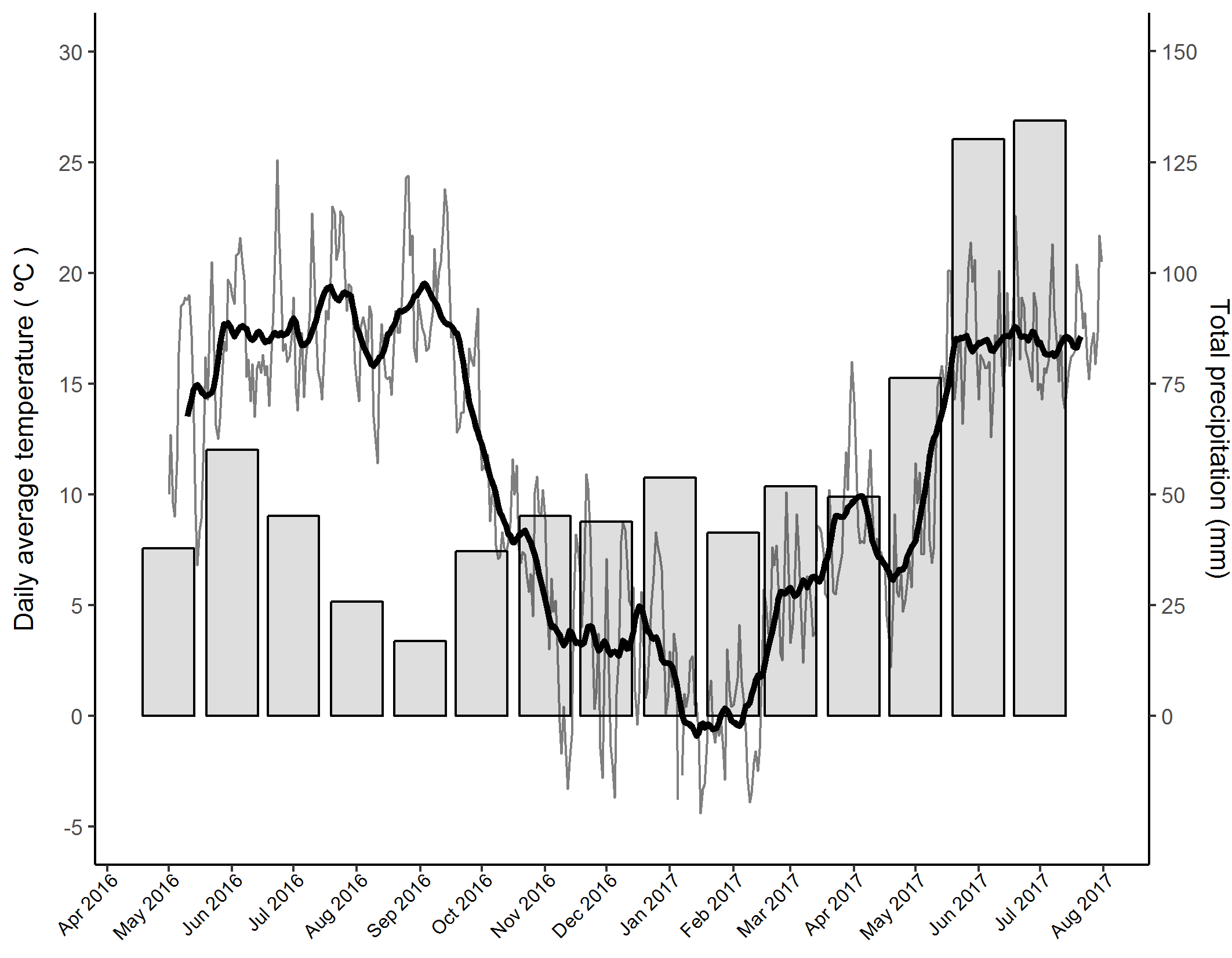


**Supplementary Figure 1.** Daily average temperature (grey line), moving average of 20 days (solid black line) and total monthly precipitation (grey bars) during this study. Data from the weather station in Wendisch Evern (53°12’49.0”N 10°28’13.1”E).


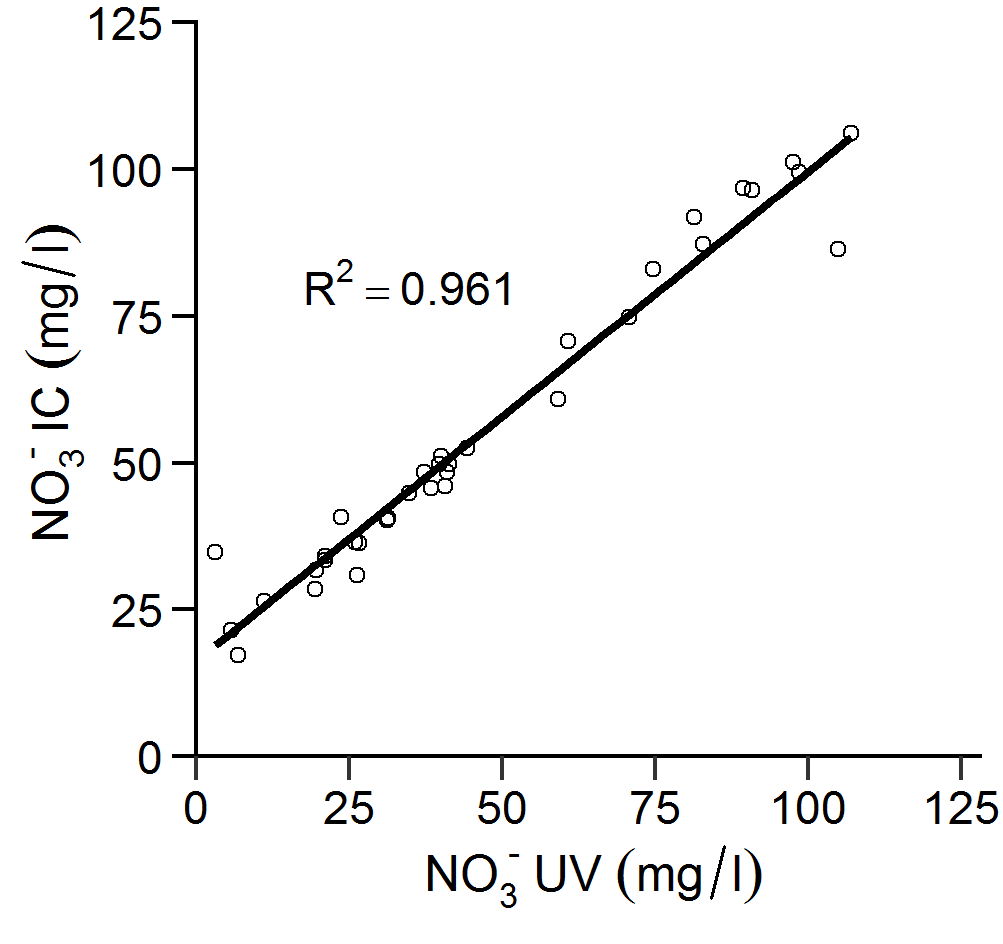


**Supplementary Figure 2.** Correlation of nitrate measured in leachate with the UV method (x-axis) and ion chromatography (y-axis) of leachate sampled at the third time point (28/11/16).


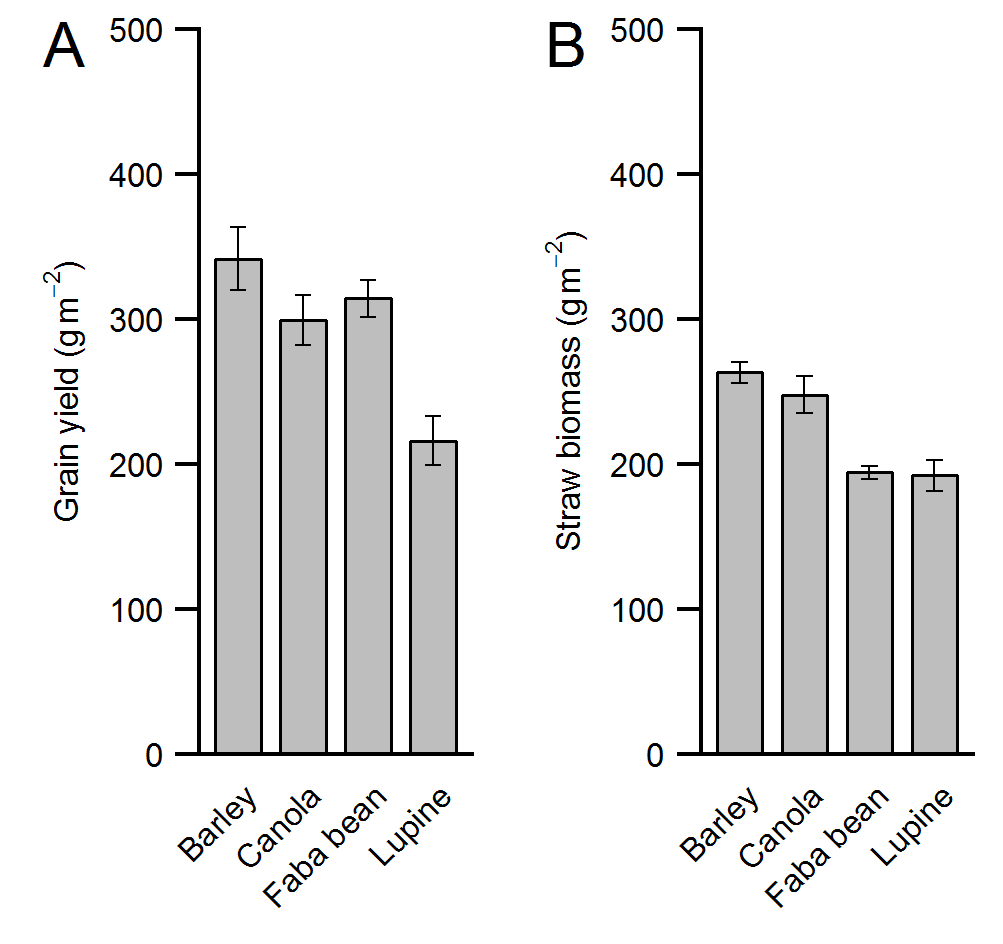


**Supplementary Figure 3.** Precrop grain yield and straw biomass at maturity. Grain yield is the total seed mass without spikes (barley) or pods (canola, faba bean, lupine) and straw biomass is all other aboveground biomass. Values are means ± se (*n=*30).

**
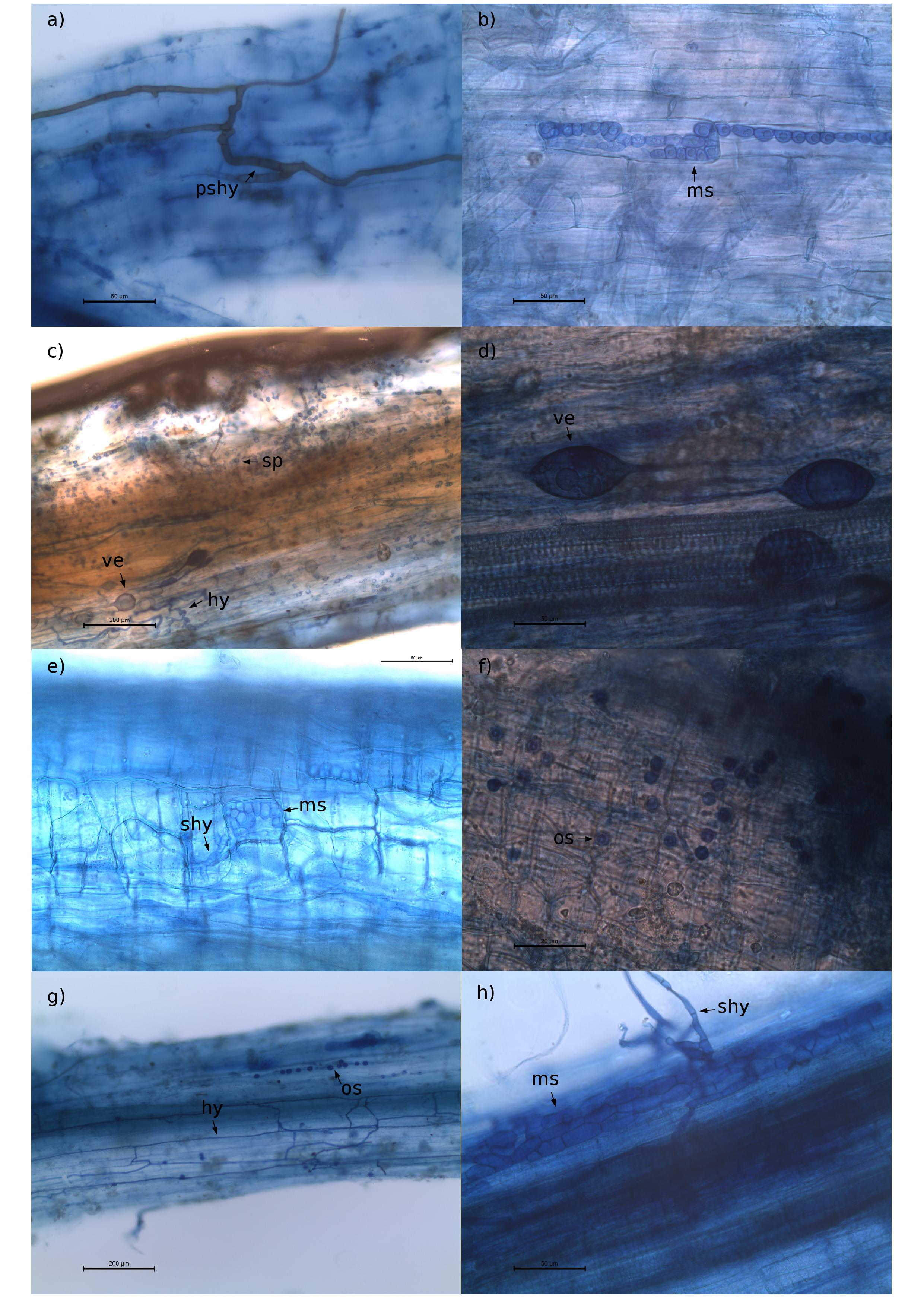
Supplementary Figure 4.** Fungal structures in precrop roots at respective harvest time. Roots were stained with Trypan blue. The following structures were observed: **(A)** Unidentified regularly pigmented septate hyphae (pshy) and **(B)** microsclerotia (ms) / alternatively moniliform cells (mc) in spring barley roots. **(C)** Glomeromycotina-like vesicules (ve), hypha (hy) and spores (sp) and **(D)** Glomeromycotina-like vesicules (ve) in faba bean roots. **(E)** Septate hyphae (shy) and microsclerotia (ms) and **(F)** Olpidium spores (op) in lupin roots. **(G)** Extracellular hyphae (hy) and Olpidium spores (os), and **(H)** microsclerotia (ms) and associated regularly septate hyphae (shy) in canola roots (composite root samples).
